# Impact of the COVID-19 pandemic on antidepressant consumption in the Central region of Portugal: interrupted time series

**DOI:** 10.1007/s00127-024-02731-0

**Published:** 2024-07-13

**Authors:** Luciana G. Negrão, Catarina Coelho, M. Margarida Castel-Branco, Isabel V. Figueiredo, Fernando Fernandez-Llimos

**Affiliations:** 1 Pharmacology and Pharmaceutical Care Laboratory, Faculty of Pharmacy, University of Coimbra, Coimbra, Portugal; 2 Administração Regional de Saúde do Centro, IP (ARSC), Coimbra, Portugal; 3 Coimbra Institute for Clinical and Biomedical Research (iCBR), Coimbra, Portugal; 4 Laboratory of Pharmacology, Department of Drug Sciences, Faculty of Pharmacy, University of Porto, Jorge de Viterbo Ferreira 228, Porto, 4050-313 Portugal; 5 Applied Molecular Biosciences Unit (UCIBIO), Porto, Portugal

**Keywords:** COVID-19, Interrupted time series analysis, Antidepressive agents, Drug utilization, Pharmacoepidemiology

## Abstract

**Purpose:**

To evaluate the impact of the pandemic on the consumption of antidepressive agents in Central Portugal.

**Methods:**

To estimate the causal effect of the pandemic an interrupted time series analysis was conducted. Data of antidepressant drugs monthly dispensed in community pharmacies between Jan-2010 and Dec-2021 were provided by the regional Health Administration. Anti-Parkinson dopaminergic agents and statins, theoretically not influenced by COVID-19 pandemics, were used as comparator series. The number of packages was converted into defined daily doses and presented as defined daily doses/1000 inhabitants/day. A Bayesian structural time-series model with CausalImpact on R/RStudio was used to predict the counterfactual. Analyses with different geographical granularity (9 sub-regions and 78 municipalities) were performed.

**Results:**

When compared to counterfactual, regional consumption non-significantly increased after the pandemic declaration, with a relative effect of + 1.30% [95%CI -1.6%:4.2%]. When increasing the granularity, differences appeared between sub-region with significant increases in Baixo Mondego + 6.5% [1.4%:11.0%], Guarda + 4.4% [1.1%:7.7%] or Cova da Beira + 4.1% [0.17%:8.3%], but non-significant variation in the remaining 6 sub-regions. Differences are more obvious at municipality level, ranging from increases of + 37.00% [32.00%:42.00%] to decreases of -11.00% [-17.00%:-4.20%]. Relative impact positively correlated with percentage of elderly in the municipality (*r* = 0.301; *p* = 0.007), and negatively with population density (*r*=-0.243; *p* = 0.032). No other predicting variables were found.

**Conclusion:**

Antidepressant consumption suffered very slight variations at regional level after the COVID-19 pandemic declaration. Analysis with higher granularity allowed identifying municipalities with higher impact (increase or decrease). The absence of clear association patterns suggests other causal hypotheses of the differences.

**Supplementary Information:**

The online version contains supplementary material available at 10.1007/s00127-024-02731-0.

## Introduction

The first cases of COVID-19 in Portugal were registered on March 2, 2020. To mitigate the transmission of the virus, Portuguese government immediately implemented severe stringent policies, such as lockdowns, workplace and school closures, and cancellation of public events, starting on March 6. These restrictive measures had consequences at health, economic, social, and demographic levels. Studies reported a rise in unemployment rates, in teleworking, as well as a reduction in the number of marriages, in tourism, and modifications in consumption patterns of alcohol beverages, illicit drugs, and medicines [1–4]. Health care systems, especially primary health care, experienced major disruptions, and reorganization all over the world, including Portugal. All elective and non-urgent services, including surgeries, vaccination, mental health, infectious diseases, oncology, nutrition, sexual, and reproductive health services, were suspended or postponed focusing on treating COVID-19 patients [3, 5].

The literature also reported a negative impact of the pandemic on mental health. People suddenly had to change the way they lived, worked, and communicated and were exposed to high levels of stress, fear, social and physical isolation, financial insecurity, and unemployment [6, 7]. Additionally, there were limitations to the practice of physical activity and on the access to mental health services. All these combined could be contributing factors to the deterioration of mental health [1, 8]. Reports alerted of an increase in mental distress symptoms in the first year of the pandemic, as well a higher risk of suicidal ideation among young people [9, 10]. Several studies conducted in Portugal described signs of psychological distress in the population, such as anxiety, depression, and post-traumatic stress disorder symptoms, as well as an increment in depression and anxiety disorder diagnoses and in psychiatric appointments in adults, children, and adolescents [1, 3, 11].

COVID-19 pandemics also affected medicine consumption in different countries, in particular psychotropic drugs, such as benzodiazepines, opioid, anti-dementia, antidepressants, and antipsychotic drugs [12–15]. A study conducted in Portugal reported an immediate significant reduction in the prescription of anxiolytics, sedatives, and hypnotics, especially in children, adolescents, and elderly women when the pandemic emerged, followed by a non-significant growth [16]. Despite the initial reduction, an increasing trend was observed in the prescription of these drugs in the first year of the pandemic, contradicting the decreasing trend observed until 2020. This study also revealed an immediate reduction in antidepressant prescription in adolescents and elderly women, as well as a decreasing trend in men in the first year of the pandemic, contrary to the pre-existing growing trend. The study found no significant changes in the consumption of antidepressants in women [16]. Another study conducted in Portugal northern region identified a regional increasing trend in psychotropic drugs between 2016 and 2021, with an increase of 9% between 2019 and 2021 [17]. The consumption of benzodiazepines and analogues had a rise of 4% between 2019 and 2021, despite the previously existing decreasing trend. As for antidepressants, there was an increase of 13% between 2019 and 2021. They also reported an increase of 6% in the diagnosis of depression in primary care, 11% of anxiety disorders, and 16.1% of sleep disorders. The authors attributed these findings to the pandemic [17].

It is important to note that Portugal has a high prevalence of psychiatric disorders, with depression being one of the main causes of disability-adjusted years, and one of the countries in Europe and the Organization for Trade and Economic Development with the highest consumption of antidepressants and benzodiazepines [18, 19]. Psychotropic drug dispensing data is a good indicator of populational mental health status, as well as of access to mental health services [20].

The aim of this study was to evaluate the impact of COVID-19 pandemic and the associated stringent policies on the consumption of antidepressant drugs in the Central region of Portugal.

## Methods

### Study design and setting

This study consisted in a retrospective observational controlled Bayesian structural time-series model of the monthly dispensing data of antidepressant drugs (ATC group N06A) prescribed in primary care centers of the Central Region Health Administration (ARSC) and dispensed in any community pharmacy between January 2010 and December 2021.

The Portuguese National Healthcare System (NHS) has universal coverage, available for all Portuguese citizens and legal residents. Health care is provided in several institutions under the Ministry of Health control, such as hospitals, local health units, and health care centers. Primary care services are provided in local units and health care centers and, at the time of the study, were organized and coordinated from a regional level. The Central region (ARSC) comprised 9 sub-regions, each of them constituted by local units and groupings of health care centers, serving a total of 78 municipalities with a total 1.6 million residents (in 2021) in a 23,600 square kilometer area, and three cities over 100.000 inhabitants. The remaining 75 municipalities have a median population of 11,283 residents (IQR 6,239:23,143). When a prescription is filled in at a community pharmacy, patients pay an out-of-pocket part, and the copayment is then reimbursed by the Government according to the reimbursement scale assigned. For antidepressant drugs, Portuguese NHS reimburses 37% of the reference price. To claim for this copayment, at the end of every month community pharmacies bill the drugs dispensed, submitting the data to the Control and Monitoring Centre of the NHS. This centre validates and keeps this information in databases. All medicines from the ATC class N, as well as the statins require a valid prescription to be dispensed and are reimbursed by the NHS. Data compiled by the Control and Monitoring Centre include the general NHS system, as well as other sub-systems (e.g., military, civil servants). Thus, this claim database constitutes a reliable source of information of dispensed drugs in Portugal.

A time series is a continuous sequence of observations collected at equally spaced time intervals. It can be interrupted by an intervention or an unscheduled event, namely a pandemic. Interrupted time series (ITS) analysis allows detecting whether the interruption event had a significant impact on the outcome under analysis, that is, an effect significantly greater than the underlying secular trend. The secular trend is the rate of change in data that precedes the event that interrupted the series. ITS is a robust study method, as it allows retrospective analysis of longitudinal data to establish causation of an event into the trend of observations. Because they use data collected in a real environment, they make it possible to assess the impact of the interrupting event on real-world healthcare practice, including medication use [21–23].

### Data collection/data sources

As control series to calculate the counterfactual series (synthetic control), we selected the time series of the dispensing data of two pharmacotherapeutic classes. Dopaminergic agents’ class (N04B) was selected as a central nervous system drug class, which should not be affected by COVID-19 pandemic, conversely to was demonstrated for other CNS drugs [13–15]. The statins group (C10A, C10B, A10BH) was the second comparator selected because they represent a highly consumed drug class, which should also be unrelated to COVID-19. None of the comparators should suffer from seasonality. To ensure that the three series presented similar seasonality, the seasonality index was calculated as the quotient between the monthly average (during the 12 years) and the overall average of each of the three series.

Monthly dispensing data for the three therapeutic classes from January 2010 to December 2021 were extracted by the ARSC from the claim files. This period allowed a pre-event series with 122 time points (January 2010 to February 2020) and 22 post event time points (March 2020 to December 2021). The literature suggested that these two periods are long enough to establish the secular trend before the pandemic (baseline), and to identify the presence of autocorrelation or seasonality [24, 25].

The data submitted by the ARSC were grouped by prescribing healthcare center, corresponding to the center where patients are allocated by residence proximity. This data comprised the number of packages prescribed in the ARSC health care centers and dispensed in any community pharmacy of the country, according to the claim database. Files included the international nonproprietary name, dosage form, strength, and package size, but no personal information. A data quality assurance process was conducted to ensure data validity and completeness. The resident population in every municipality at the end of each year was obtained from the PORDATA database (www.pordata.com*).* To avoid abrupt jumps in the population time series, yearly variations were equally distributed during the 12-month interval (i.e., adding a 1/12 monthly aliquot).

### Data analysis

The number of packages was converted into defined daily doses (DDD) using the WHO Collaborating Centre for Drug Statistics Methodology. The DDD of each drug was obtained from the ATC/DDD website (https://www.whocc.no/atc_ddd_index/). In the case of pirlindole, which does not have an ATC code or DDD assigned, we used the average daily dose for the main indication in an adult. The DDD modifications from 2005 to 2020 was also consulted to obtain information on possible changes in DDD during the study timeframe. Whenever changes occurred, the most current DDD assigned was used, as recommended elsewhere [26]. So, the primary outcome for the ITS was the number of DDD of N06A drugs monthly dispensed. As recommended, DDDs are presented as DDD/1000 inhabitants/day (DID). DID gives an estimative of the population under study (in permillage) that is daily using a given drug.

**Fig. 1:**
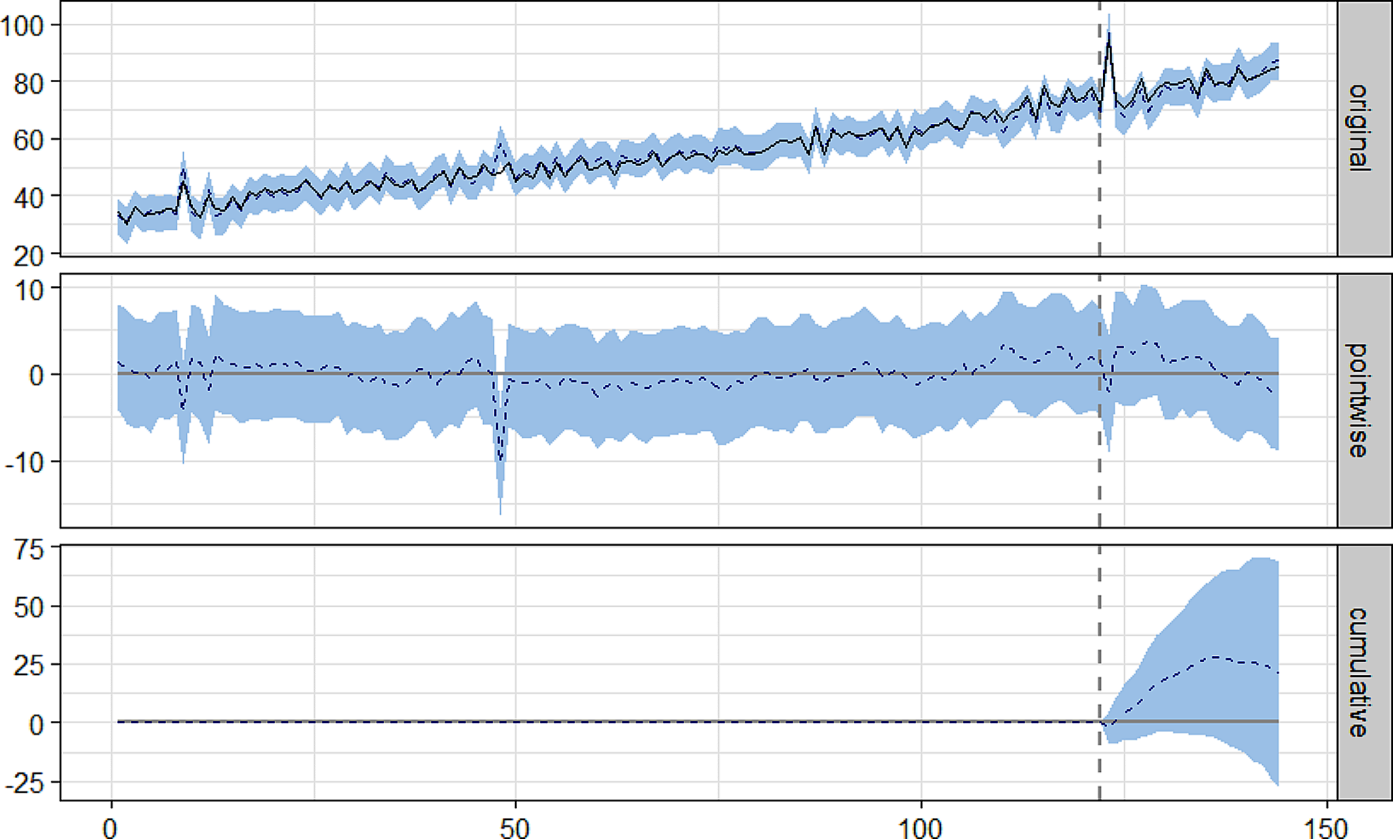
CasualImpact analysis plot of antidepressant consumption in DDD/1000inh/day (DID) in the Portugal Central region between 2010 and 2021

To estimate the causal impact of COVID-19 pandemic in antidepressant consumption, we used a Bayesian structural time-series (diffusion-regression state-space) model. The analysis was made using the CasualImpact package [24] on R/RStudio (Posit, Boston, MA). This model uses the comparator time series (N04B and statins), and the time series under study itself (N06A) to construct a synthetic control and to predict the counterfactual. This approach accommodates multiple sources of variation, such as local trends, seasonality, and autocorrelation. The causal impact is the difference between the observed time series (actual antidepressant consumption after the pandemic) and the counterfactual time series inferred (estimated consumption that could have been observed if the pandemic never took place). The CausalImpact package performs a statistical analysis (posterior inference) and calculates the posterior predictive expectation of the counterfactual with pointwise 95% posterior probability intervals and 95% credible intervals of the cumulative impact [24]. To facilitate the interpretation of the statistics and to identify potential join points, CasualImpact provides a three-part figure with a vertical grey dashed line represents the interruption of the time series by the COVID-19 pandemic (February 2020) and three plots (Fig. 1):

- Original plot: The solid black line depicts the actual antidepressant consumption during the time period under study. The blue dashed line depicts the counterfactual predictions (synthetic control), i.e., antidepressant consumption expected if the pandemic had not occurred. The overlapping of the two lines after the interruption indicates that there was no impact caused by the pandemic. The blue shaded area represents the 95% credible interval (95%CI).
- Pointwise plot: The blue dashed line is the difference between the observed consumption data and the counterfactual predictions. It stands for the inferred causal impact of the pandemic. If the line is close to zero, it means there was no causal impact. The blue shaded area represents the 95% credible interval.
- Cumulative plot: The blue dashed line shows, for each month, the summed effect of the pandemic up to that month. The blue shaded area represents the 95% credible interval. When the 95% credible interval crosses the zero line, it means there was a non-significant effect.

The impact analysis was conducted with three levels of granularity: the entire region of the ARSC, the 9 sub-regions and the 78 municipalities.

To ascertain whether or not there exist any association between the influence of COVID-19 and socio-demographic characteristics, a bivariate analysis (i.e., Student’s t tests) was conducted to study the association between having an increase of antidepressant consumption in the post-COVID-19 period and the following factors: illiteracy rate in both sexes in 2011, per capita purchasing power indicator in 2019, antidepressant consumption in 2021 (in DID), population (2021), percentage of the elderly population (2021), inhabitants per pharmacy (2021), total number of inhabitants per health center and population density in 2021. Also, a Pearson’s correlation analysis was also conducted between these factors and the relative effect in percentage. Statistical significance was considered when p-value was *p* < 0.05.

### Ethics approval

Dispensing data were provided by the ARSC aggregated and anonymously, which made it impossible to link the obtained data to individual persons. Thus, protection and confidentiality of personal data was guaranteed and informed consent does not apply to this context. This study was evaluated and approved by the ARSC Ethics Committee (ARSC 53/2021).

## Results

Overall, antidepressant consumption in the Portugal Central region presented an increasing trend during all the study period from the 38 DID in 2010 to the 73 DID in 2019 and 81 DID in 2021. Yearly regression trend shows a 4.02 DID slope with *R* = 0.984. Neither antidepressant time series not comparator series presented a relevant seasonality, with monthly seasonality indexes ranging between 0.91 and 1.05 in antidepressants and an almost identical profile among the three series throughout the year (Supplementary File 1). Selective serotonin reuptake inhibitors (N06AB) were the most prescribed class during all the period, varying from 68 to 70% of total antidepressant DDD, followed by other antidepressants class (N06AX) that ranged from 24 to 27%. Important differences in antidepressant consumption existed among municipalities during this period, with intervals ranging from 18 to 71 DID in 2010 and from 38 to 157 DID in 2021 (Supplementary File 2).

Figure 1 depicts the ITS analysis of the entire ARSC, where a non-significant increase of + 1.20% [95%CI -1.6%:3.9%] in antidepressant consumption occurred after the COVID-19 pandemic declaration, when compared to the counterfactual.

**Fig. 2:**
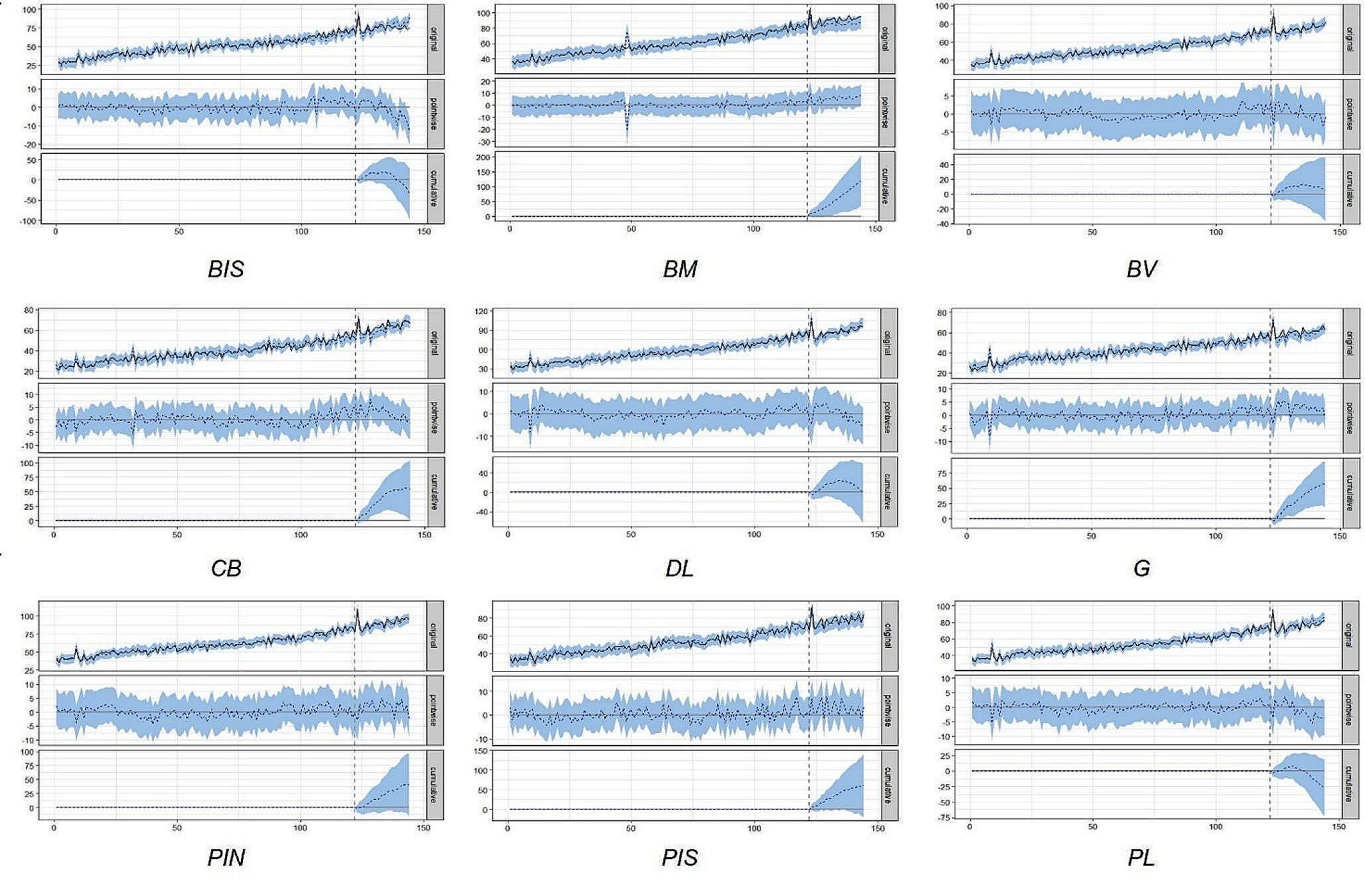
ITS CausalImpact analysis plots of antidepressant consumption (DID) in the ARSC sub-regions of the Portugal Central region (ACeS/ULS) between 2010 and 2021. BM: Baixo Mondego; BV: Baixo Vouga; CB: Cova da Beira; DL: Dão Lafões; PIN: Pinhal Interior Norte; PL: Pinhal Litoral; BIS: Beira Interior Sul; PIS: Pinhal Interior Sul; G: Guarda

Table 1 sums up the posterior inference results of the impact analysis of the entire ARSC and of its 9 sub-regions (ACeS/ULS). There was a significant increase in three sub-regions: Baixo Mondego + 6.5% [1.4%:11%], Guarda + 4.4% [1.1%:7.7%] and Cova da Beira + 4.1% [0.17%:8.3%], but non-significant variation in the remaining 6 sub-regions Fig. 2 shows the ITS analysis plots for all the 9 sub-regions of the ARSC.

**Table 1:** CausalImpact analysis of antidepressant consumption in DDD/1000inh/day (DID) in Portugal Central Region and sub-regions between 2010 and 2021

	Actual DID	Predictive DID	95% CI	Absolute effect	95% CI	Relative effect	95% CI	*p*-value
BIS	76	77	74:80	-1.60	-4.4:1.2	-2.10	-5.5%:1.7%	0.127
BM	91	85	81:89	5.60	1.2:9.1	6.50%	1.4%:11%	**0.007**
BV	75	75	73:77	0.24	-1.9:2.3	0.35%	-2.4%:3.3%	0.409
CB	63	61	58:63	2.50	0.11:4.9	4.10%	0.17%:8.3%	**0.020**
DL	88	88	85:91	-0.056	-2.7:2.9	0.034%	-3.0%:3.4%	0.500
G	61	58	57:60	2.60	0.69:4.3	4.40%	1.1%:7.7%	**0.004**
PIN	91	89	87:92	1.80	-0.69:4.3	2.00%	-0.75%:5.0%	0.084
PIS	78	76	72:79	2.80	-0.10:6.2	3.70%	-1.3%:8.6%	0.076
PL	76	78	76:80	-1.30	-3.3:0.79	-1.60	-4.1%:1.0%	0.110
Region	80	79	77:81	0.95	-1.3:3.1	1.30%	-1.6%:4.2%	0.200

*95%CI: 95% credible interval; BM: Baixo Mondego; BV: Baixo Vouga; CB: Cova da Beira; DL: Dão Lafões; PIN: Pinhal Interior Norte; PL: Pinhal Litoral; BIS: Beira Interior Sul; PIS: Pinhal Interior Sul; G: Guarda*

**Fig. 3:**
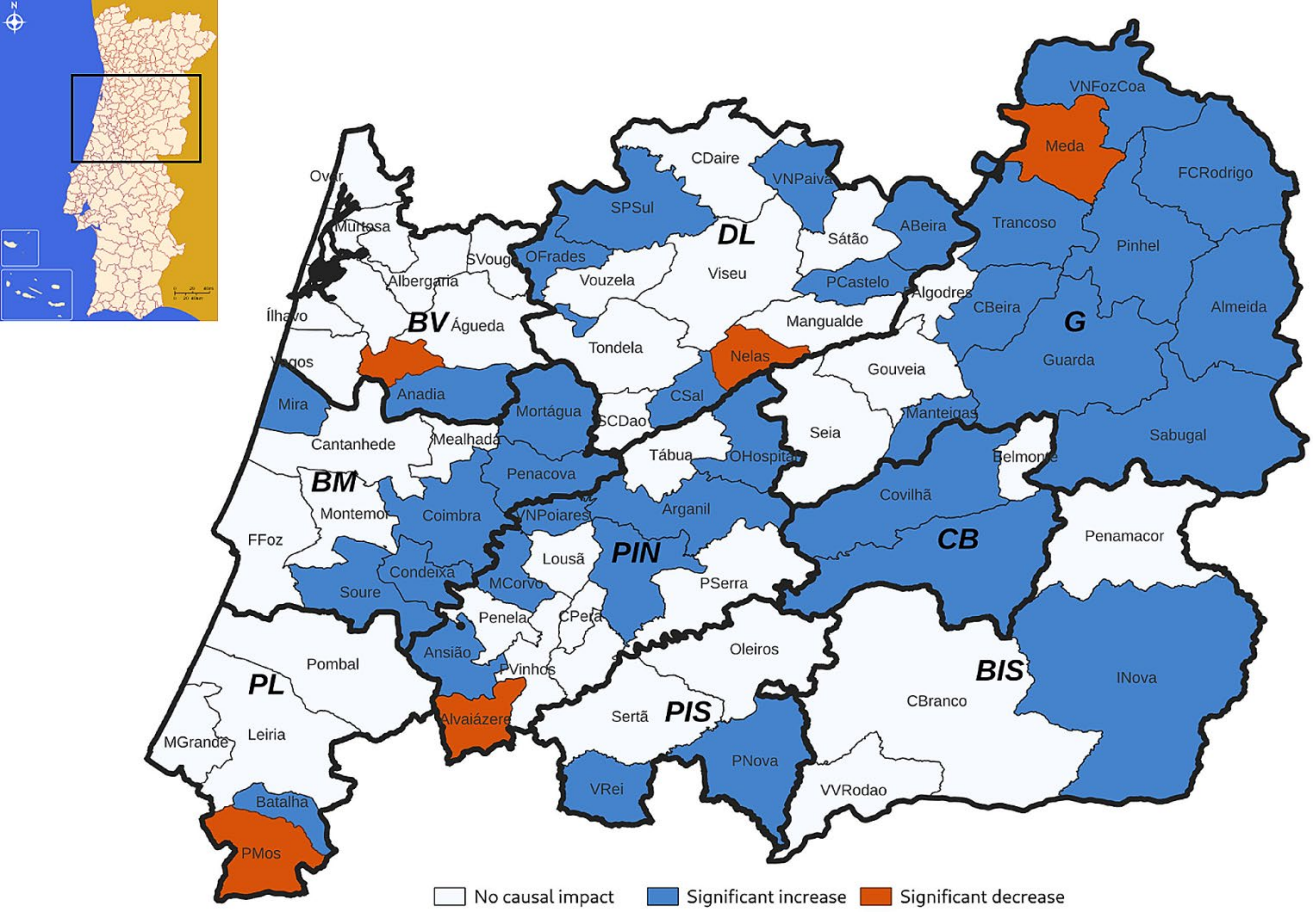
Geographical distribution of the causal impact of the COVID-19 pandemic in Portugal Central region at municipality level. BM: Baixo Mondego; BV: Baixo Vouga; CB: Cova da Beira; DL: Dão Lafões; PIN: Pinhal Interior Norte; PL: Pinhal Litoral; BIS: Beira Interior Sul; PIS: Pinhal Interior Sul; G: Guarda

Impact analysis at municipality level revealed a significant increase in antidepressant consumption in 34 municipalities and a significant decrease in 5 municipalities. Some municipalities had sharp increases, such as Almeida + 37.00% [95%CI 32.00%:42.00%], Arganil + 17.00% [95%CI 13.00%:21.00%] or Manteigas + 19.00% [95%CI 14.00%:25.00%], as others had sharp reductions, such as Nelas − 10.00% [95%CI -15.00%:-5.50%] or Mêda -11.00% [95%CI -17.00%:-4.20%] (Supplementary File 3). Figure 3 shows the geographical distribution of the causal impact analysis of COVID-19 pandemic on antidepressant consumption in the municipalities of ARSC.

The bivariate analysis revealed no significant differences in municipalities that had a significant increase in antidepressant consumption when compared to those with no increase, for the following covariables: antidepressant consumption in 2021 (*p* = 0.225), population in 2021 (*p* = 0.136), percentage of elderly population (> 65 years old) in 2021 (*p* = 0.062), per capita purchasing power indicator in 2019 (*p* = 0.155), inhabitants per pharmacy in 2021 (*p* = 0.208), total inhabitants per health center in 2021 (*p* = 0.453), or illiteracy rate (%) in both sexes in 2011 (*p* = 0.081). However, a significant difference (*p* = 0.029) was found for population density: there was an increase in municipalities with a mean population density of 62.4 inhabitants/km^2^ (SD = 76.6), while municipalities in which there was no increase had on average 112.8 inhabitants/km^2^, (SD = 112.8).

Causal relative impact, expressed in percentage of differences between actual and contrafactual series, did not correlate with antidepressant consumption in 2021 (*p* = 0.571), population in 2021 (*p* = 0.079), per capita purchasing power indicator in 2019 (*p* = 0.066), inhabitants per pharmacy in 2021 (*p* = 0.055), total inhabitants per health center in 2021 (*p* = 0.544) or illiteracy rate (%) in both sexes in 2011 (*p* = 0.111). Conversely, relative impact negatively correlated with population density in the municipality (*r*=-0.243; *p* = 0.032), and positively with percentage of elderly population (*r* = 0.301; *p* = 0.007).

## Discussion

Using a robust Bayesian method to perform ITS analysis, we could find that, conversely to what is expected, antidepressant consumption in Portugal central region slightly increased but with no statistical significance after the COVID-19 pandemic declaration. Both in the regional and the sub-regional analysis a transient rise in antidepressant drugs dispensing was observed at the initial phases of pandemic period. But considering the entire post-pandemic period, antidepressant consumption quickly returned to the trend observed in the pre-pandemic period, except for Baixo Mondego and Guarda. However, increasing the granularity of the analysis, statistically significant changes were observed in 39 out of the 78 municipalities, with 34 municipalities showing a positive impact and 5 a negative impact of COVID-19. Some of these municipalities presented an important increase of more than 20%, and some others an important decrease of about 10%.

The immediate and sharp peak observed right after the time series interruption may be associated to stockpiling practices that occurred in the initial month of the COVID-19 pandemic lockdown [20, 27]. This is also reported in studies in Poland, Canada, and United States of America, where antidepressant prescription or dispensing had a shift right after the pandemic emerged [28, 29], but no significant impact on trends or consumption patterns [27, 30].

In Portugal, a study by Estrela et al. [16]. described similar overall results to our study. Using a segmented regression ITS analysis, the authors did not detect a significant impact of the pandemic on the consumption of antidepressants when analyzing total dispensing data. However, when the analysis was stratified by sex and age group, they found a statistically significant reduction in the consumption of these drugs in the male population over the age of 8, and no significant changes in the consumption by women. Likewise, when we increased the granularity level of our analysis, we were able to identify considerable differences in municipalities with different characteristics. The increase of antidepressant consumption detected in almost half of the ARSC municipalities is in agreement with the published literature on the impact of the pandemic on the mental health of the Portuguese population. There are several studies that indicate a deterioration in mental health, namely an increase in symptoms of depression and anxiety, psychological distress, and post-traumatic stress disorders, not only in the initial phase of the pandemic, but also throughout its course [3, 31–33]. In fact, one of these studies reports higher levels of depression and stress symptoms during the second confinement, when compared to the beginning of the pandemic [31]. However, we also identified significant decreases in 5 municipalities. The discrepancies in the relative impact identified in this study, as well as in the total consumption of antidepressants among municipalities during the study period, may highlight possible differences in equity and access to mental health services among the Portuguese population and which are referred to in the National Mental Health Program 2007–2016.

A significant rise in antidepressant consumption was observed in municipalities with lower density population and with a higher percentage of elderly people, which mostly correspond to the rural and mountain sub-regions. This might be a consequence of the social isolation that occurred during the lockdown periods, that may have exacerbated feelings of loneliness in a more vulnerable population, worsening the mental health of the population in these municipalities, and leading to an increase of antidepressant consumption [34].

### Strengths and limitations

One of the strengths of this study is the robust method used to perform the analyses. We conducted an ITS analysis using a Bayesian structural time-series model to predict the counterfactual and to infer the causal impact of the pandemic. This model offers advantages over classical regression approaches, such as segmented regression approaches, as these often assume linear behavior in the variables. Additionally, regression approaches are not suitable for drawing counterfactual predictions for long periods when dealing with time series with autocorrelation or non-linear variation. Another strong point of this method is the use of comparator groups to calculate the synthetic control and to predict the counterfactual. We also used a long time series, which allowed the model to identify with greater precision the presence of potential sources of biases (secular trend, autocorrelation or seasonality) in the data in the period before the interruption. Furthermore, it is possible to verify the validity of our analysis, as the synthetic control calculated by the model almost overlaps with the time series of real antidepressant consumption throughout the pre-event time period (baseline), which validates the counterfactual forecast calculated. Another strength of this study is the high level of granularity of the analyses (region, sub-regions, and municipalities). Our findings reinforced the importance of increasing the granularity of pharmacoepidemiologic analyses.

Nevertheless, this study also has some limitations. Our analysis included data for the 22 months after lockdown, but the effects of COVID-19 may exceed this period, which may justify additional analyses of a longer period. Data analyzed does not include drugs that have been provided without a medical prescription (not legal for these groups), consumed during hospital care, or prescribed by private health services and entirely supported by the patients, but the proportion of antidepressants (like other ambulatory psychotropic drugs) acquired outside the NHS and the subsystems is negligible. We had no access to the information about the population per health center, so we were unable to further increase the granularity to a health care center level. Since we did not have access to information on diagnoses, individual characteristics, or off-label use of medications, it is not possible to identify situations in which antidepressant drugs were not prescribed for the treatment of depression. Additionally, it is not possible to know whether all prescribed and dispensed drugs were actually consumed by patients.

## Conclusion

The consumption of antidepressant drugs in the Central region of Portugal suffered very slight not significant variations at the regional level after the COVID-19 pandemic declaration in March 2020. However, analysis with higher granularity made it possible to identify municipalities with significant causal impact (increase or decrease). The absence of clear association patterns forces us to think about other causal hypotheses for the municipal differences. Our study highlights discrepancies in the consumption of antidepressant drugs and in the impact of the pandemic between municipalities of the Central region of Portugal.

## Electronic supplementary material

Below is the link to the electronic supplementary material.

Supplementary Material 1

Supplementary Material 2

Supplementary Material 3

## Data Availability

No datasets were generated or analysed during the current study.
