# Supplementary material for: Impact of the COVID-19 pandemic on antidepressant consumption in the Central region of Portugal: interrupted time series
Source: Soc Psychiatry Psychiatr Epidemiol. 2024 Jul 13;60(3):621–9. doi: 10.1007/s00127-024-02731-0 (PMC11870879; doi:10.1007/s00127-024-02731-0)

Supplementary 1

Seasonality index of the three time-series,

$$SI_{(m)} = \frac{average.consumption_{(m)}}{average.consumption}$$

Month	Average DDD/1000inh/day			Seasonality index		
	N06A	N04B	Statins	N06A	N04B	Statins
1	55.85	0.76	96.26	0.99	0.99	0.98
2	51.27	0.70	88.74	0.91	0.90	0.90
3	57.91	0.79	100.73	1.02	1.03	1.03
4	54.08	0.73	94.26	0.95	0.96	0.96
5	56.76	0.77	98.50	1.00	1.00	1.00
6	54.79	0.74	95.24	0.97	0.96	0.97
7	58.81	0.80	101.89	1.04	1.04	1.04
8	55.95	0.77	95.95	0.99	1.00	0.98
9	57.77	0.81	99.37	1.02	1.05	1.01
10	59.59	0.81	102.66	1.05	1.05	1.05
11	58.13	0.76	100.70	1.03	0.99	1.03
12	58.84	0.79	103.99	1.04	1.02	1.06

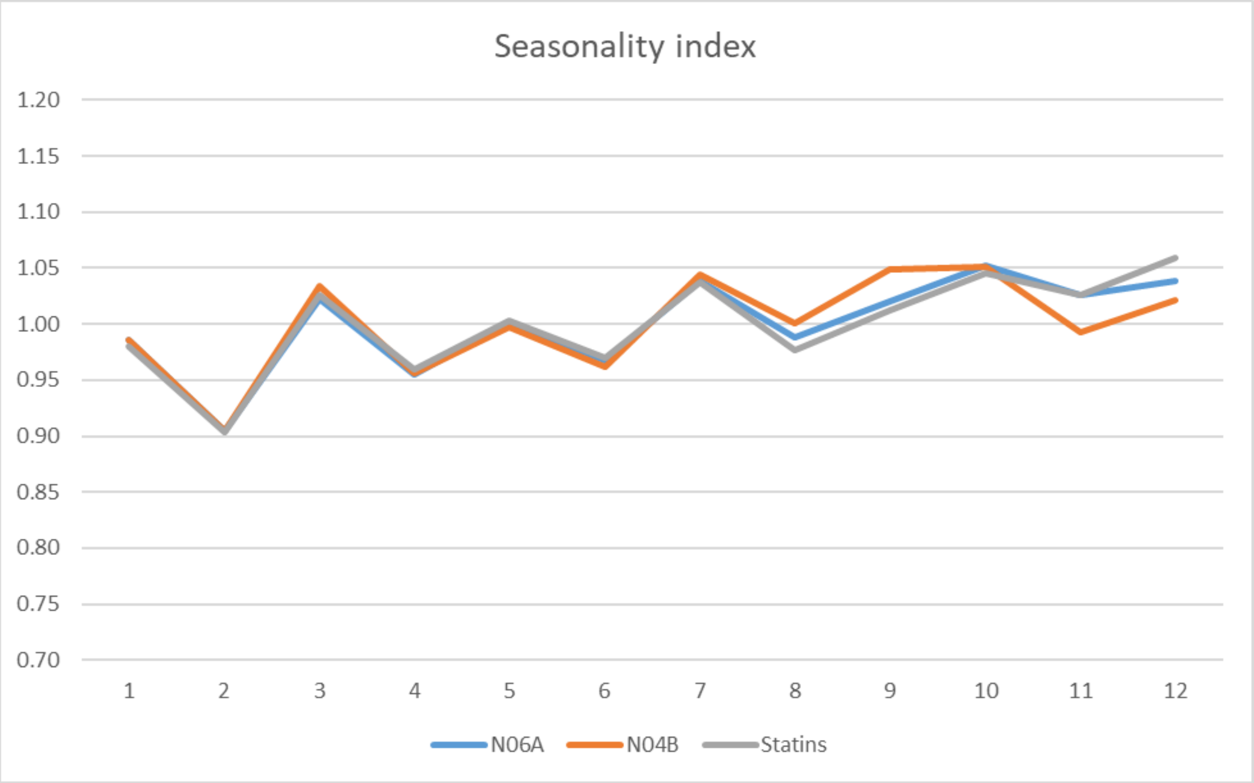

Supplement: Supplementary file 1 — Supplementary Material 1 [file 127_2024_2731_MOESM1_ESM.pdf]
