# Supplementary material for: Impact of the COVID-19 pandemic on antidepressant consumption in the Central region of Portugal: interrupted time series
Source: Soc Psychiatry Psychiatr Epidemiol. 2024 Jul 13;60(3):621–9. doi: 10.1007/s00127-024-02731-0 (PMC11870879; doi:10.1007/s00127-024-02731-0)
Supplement: Supplementary file 2 — Supplementary Material 2 [file 127_2024_2731_MOESM2_ESM.pdf]

Supplementary 1

Annual consumption of antidepressants in DDD/1000 inhabitants/day between 2010 and 2021 in municipalities of the Central Health Administration , Portugal.

Sub-re-gion	Municipality	2010	2011	2012	2013	2014	2015	2016	2017	2018	2019	2020	2021
BM	Cantanhede	43.29	44.54	46.15	47.93	50.63	54.33	58.81	65.26	71.41	80.56	87.22	95.61
	COIMBRA	32.09	40.34	44.71	50.08	53.07	56.80	60.78	65.99	69.76	76.89	84.33	83.75
	Condeixa-a-Nova	42.69	47.06	50.24	52.99	58.95	60.51	68.56	71.17	74.63	81.83	95.58	106.56
	Figueira da Foz	37.94	43.32	45.91	49.01	52.05	53.26	56.64	61.58	65.93	74.91	79.63	84.19
	Mealhada	36.40	42.25	45.79	52.81	54.44	56.41	59.96	70.21	73.30	76.80	83.05	90.20
	Mira	33.78	36.54	38.02	38.36	44.15	50.19	56.66	60.48	66.70	70.94	81.94	84.48
	Montemor-o-Velho	39.46	41.40	46.54	50.32	54.92	56.82	59.08	65.50	70.07	77.68	85.03	93.69
	Mortágua	62.67	70.59	74.84	75.87	81.29	80.18	79.52	85.93	96.37	103.74	108.23	110.24
	Penacova	44.73	49.18	56.87	62.92	62.52	63.26	68.17	75.27	79.46	88.36	94.29	107.76
	Soure	40.81	46.30	52.23	58.56	65.47	67.61	72.71	82.55	87.03	98.48	109.36	116.31
BV	Águeda	42.59	43.14	47.43	48.38	52.33	53.89	58.09	61.13	62.95	68.43	72.69	74.89
	Albergaria-a-Velha	41.24	44.17	45.68	46.78	50.29	51.39	54.80	59.49	62.69	72.76	74.46	80.16
	Anadia	43.92	46.00	46.67	49.64	51.82	51.09	57.71	61.47	64.20	73.01	78.39	81.37
	Aveiro	35.48	40.58	43.80	48.25	47.87	48.65	55.53	58.79	61.80	67.83	69.93	71.82
	Estarreja	27.54	29.78	30.82	33.16	31.52	32.93	37.85	40.79	46.06	55.37	59.62	65.09
	Ílhavo	38.62	41.85	44.51	45.25	50.09	51.29	55.01	57.64	62.18	69.14	73.90	77.19
	Murtosa	24.12	25.83	26.77	29.19	27.45	27.60	33.64	40.51	48.76	61.71	62.95	65.67
	Oliveira do Bairro	40.99	43.32	44.16	49.97	49.72	50.81	56.27	61.30	61.21	67.15	67.41	70.64
	Ovar	34.32	41.22	44.13	50.03	47.20	56.13	58.05	63.35	67.00	72.72	76.60	80.12
	Sever do Vouga	44.18	47.79	51.16	54.43	58.01	61.03	65.61	68.87	71.67	82.52	88.85	90.05
	Vagos	36.95	39.66	40.70	43.36	43.96	46.53	49.20	52.30	55.90	62.20	67.18	70.40
CB	Belmonte	29.52	32.76	38.60	42.73	48.19	46.39	52.85	57.04	61.73	65.44	77.90	86.14
	Covilhã	23.11	26.74	29.38	30.87	33.50	34.49	37.84	39.74	40.88	46.21	54.05	59.31
	Fundão	25.76	30.14	33.29	35.93	36.97	38.64	41.08	46.57	51.07	57.85	64.86	69.04
DL	Aguiar da Beira	51.06	38.55	47.85	52.90	55.29	55.79	62.27	66.02	70.41	89.58	89.78	87.15
	Carregal do Sal	43.59	45.00	47.30	54.08	57.50	62.53	66.49	70.10	75.60	79.45	86.36	95.99

Sub-re-gion	Municipality	2010	2011	2012	2013	2014	2015	2016	2017	2018	2019	2020	2021
	Castro Daire	29.73	35.10	37.38	40.08	46.14	48.66	55.81	58.89	65.74	73.19	80.13	84.94
	Mangualde	33.77	38.45	42.67	48.39	53.16	56.08	64.40	66.57	71.87	80.13	85.56	89.71
	Nelas	28.38	30.68	32.64	38.94	44.17	52.35	58.73	66.29	70.52	81.33	89.39	92.56
	Oliveira de Frades	32.01	35.92	40.36	44.87	49.20	48.20	54.15	57.07	63.71	70.32	76.05	83.73
	Penalva do Castelo	21.94	22.09	25.64	31.96	38.55	44.96	48.19	50.19	57.65	64.73	68.55	73.79
	Santa Comba Dão	43.63	51.74	59.41	64.43	74.62	79.08	81.97	89.10	93.80	106.95	119.32	129.32
	São Pedro do Sul	45.22	49.62	50.17	53.32	58.16	56.80	64.82	68.02	71.61	81.69	93.83	100.83
	Sátão	35.40	41.20	42.93	48.75	53.86	53.20	57.16	69.59	71.13	75.22	73.13	79.05
	Tondela	44.12	48.68	48.88	55.32	61.65	69.16	75.28	81.49	87.79	97.81	102.80	112.22
	Vila Nova de Paiva	42.83	52.41	56.53	59.80	64.31	70.06	78.50	83.87	91.80	96.02	97.36	101.54
	Viseu	28.69	33.73	35.70	39.37	43.42	45.61	49.70	53.50	57.94	67.83	71.14	75.45
	Vouzela	40.12	47.95	49.30	56.34	62.89	74.06	82.30	91.25	92.86	104.28	112.39	118.10
PIN	Alvaiázere	40.07	44.97	51.05	60.01	59.82	62.95	61.37	69.91	75.74	80.64	86.48	93.39
	Ansião	44.03	47.48	48.15	49.51	50.43	54.48	60.64	73.27	79.34	87.96	92.92	102.69
	Arganil	38.22	40.43	41.54	40.86	38.89	39.53	41.18	49.21	52.50	54.90	66.09	68.34
	Castanheira de Pera	46.41	56.87	59.62	52.31	59.38	60.69	68.15	82.34	93.47	105.03	119.43	113.91
	Figueiró dos Vinhos	56.29	64.33	68.31	65.71	74.67	76.24	79.79	80.55	89.57	98.49	110.32	121.97
	Góis	43.69	50.05	51.44	54.60	62.31	60.82	61.70	61.48	64.67	68.00	76.17	74.90
	Lousã	42.03	52.29	56.49	62.56	69.47	69.38	68.54	73.48	76.19	83.23	88.37	96.94
	Miranda do Corvo	38.23	43.41	45.87	51.01	51.04	56.18	59.78	63.73	66.51	72.68	84.40	95.10
	Oliveira do Hospital	25.11	26.36	30.52	34.49	37.15	38.71	40.17	43.77	49.02	56.69	62.86	72.55
	Pampilhosa da Serra	70.78	72.33	81.38	79.50	80.09	81.88	80.72	84.60	90.63	85.49	88.19	84.48
	Pedrógão Grande	65.70	78.90	77.29	77.30	81.02	89.78	93.03	109.59	125.07	138.96	141.60	156.60
	Penela	57.01	58.12	62.99	58.62	66.82	78.50	84.13	89.06	91.63	101.49	110.99	115.25
	Tábua	42.24	44.35	50.91	54.71	57.44	62.66	68.75	75.15	78.10	90.06	96.02	99.87
	Vila Nova de Poiares	33.73	37.20	40.93	49.17	54.33	56.90	56.95	62.06	60.24	69.04	78.87	80.58
PL	Batalha	40.19	44.92	49.08	51.51	52.72	60.38	64.14	69.15	72.20	75.88	79.69	88.78
	Leiria	35.15	40.49	43.47	45.74	47.91	53.31	56.16	61.22	65.22	72.96	77.51	79.30
	Marinha Grande	32.21	31.47	32.83	35.26	36.44	38.22	41.07	45.62	48.97	54.00	57.32	59.16
	Pombal	40.36	43.27	45.46	47.58	50.17	51.60	55.27	60.34	61.92	70.19	71.96	76.83

Sub-region	Municipality	2010	2011	2012	2013	2014	2015	2016	2017	2018	2019	2020	2021
	Porto de Mós	41.75	48.73	50.19	51.60	52.58	58.76	58.07	63.94	67.42	73.85	76.92	78.23
BIS	Castelo Branco	30.75	36.44	40.38	45.22	47.81	51.77	52.27	55.85	61.66	68.33	73.74	75.04
	Idanha a Nova	28.94	37.59	35.99	37.74	43.91	44.60	46.15	51.48	55.51	61.47	72.38	66.07
	Penamacor	24.60	31.48	38.08	42.25	43.91	43.57	47.93	51.02	52.95	69.54	73.67	73.38
	Vila Velha Rodão	39.23	38.11	43.00	44.97	53.12	56.29	58.88	61.61	68.63	80.40	79.95	82.71
PIS	Oleiros	33.22	41.52	41.54	46.43	50.46	57.91	62.94	67.59	76.36	87.01	90.53	89.29
	Proença a Nova	43.91	47.26	51.44	53.79	51.33	65.10	63.90	68.27	74.11	82.17	93.26	101.53
	Sertã	28.06	29.00	33.21	37.93	41.77	43.47	43.16	47.76	51.25	58.48	63.22	64.79
	Vila de Rei	41.42	47.44	51.96	60.57	56.03	55.67	51.93	61.94	58.61	55.45	62.50	74.38
G	Almeida	21.90	29.37	31.79	33.11	35.78	40.21	42.20	44.54	51.13	60.36	76.31	80.36
	Celorico Beira	27.72	33.62	38.88	39.37	37.88	38.43	37.65	42.63	46.69	48.13	53.73	65.91
	Fornos Algodres	41.77	49.23	51.58	53.21	57.59	59.31	61.25	63.84	65.05	69.07	73.39	77.18
	Figueira Castelo Rodrigo	28.72	34.59	36.49	40.51	39.62	38.95	35.18	30.82	31.63	36.85	37.58	46.14
	Gouveia	36.66	44.96	46.86	49.45	52.32	55.87	58.43	63.42	67.04	71.72	72.80	68.59
	Guarda	18.18	23.02	24.21	25.62	28.96	31.76	32.26	34.54	35.45	39.88	42.24	42.49
	Manteigas	46.00	63.81	70.57	68.08	54.36	53.75	56.32	68.91	81.06	86.58	92.53	111.24
	Meda	20.08	26.81	28.64	34.55	31.12	37.52	47.98	59.28	63.05	72.36	80.84	80.22
	Pinhel	28.23	33.44	32.62	41.19	41.01	45.95	49.42	54.84	54.41	60.61	69.79	81.56
	Sabugal	22.52	23.74	23.65	21.98	23.82	26.57	27.48	27.90	30.58	32.70	38.72	37.50
	Seia	33.81	39.52	43.15	47.60	49.88	57.58	59.16	63.77	63.47	67.74	74.85	75.90
	Trancoso	24.72	30.16	31.82	34.13	33.78	37.45	38.49	38.84	42.48	47.94	50.82	57.85
	Vila Nova de Foz Côa					5.31	54.81	56.87	63.47	66.09	66.76	68.65	70.60
	Min	18.18	22.09	23.65	21.98	5.31	26.57	27.48	27.90	30.58	32.70	37.58	37.50
	Percentile 25%	29.52	34.59	38.02	40.51	43.91	46.06	49.49	55.09	58.10	65.77	70.23	73.48
	Median	38.22	41.52	44.71	48.75	50.84	54.41	57.88	62.70	66.01	72.74	78.15	81.46
	Percentile 75%	42.83	47.44	50.24	53.79	57.49	60.47	64.08	69.48	74.50	82.08	89.25	95.48
	MAX	70.78	78.90	81.38	79.50	81.29	89.78	93.03	109.59	125.07	138.96	141.60	156.60

95%CI: 95% credible interval; BM: Baixo Mondego; BV: Baixo Vouga; CB: Cova da Beira; DL: Dão Lafões; PIN: Pinhal Interior Norte; PL: Pinhal Litoral; BIS: Beira Interior Sul; PIS: Pinhal Interior Sul; G: Guarda.
