# Supplementary material for: Impact of the COVID-19 pandemic on antidepressant consumption in the Central region of Portugal: interrupted time series
Source: Soc Psychiatry Psychiatr Epidemiol. 2024 Jul 13;60(3):621–9. doi: 10.1007/s00127-024-02731-0 (PMC11870879; doi:10.1007/s00127-024-02731-0)
Supplement: Supplementary file 3 — Supplementary Material 3 [file 127_2024_2731_MOESM3_ESM.pdf]

Supplementary File 3

CausalImpact analysis of antidepressant consumption in the municipalities of the Central Health Administration , Portugal between 2010 and 2021

Sub-regions	Municipality	Relative Effect	95% CI	p-value
BM	Cantanhede	0.64%	-3%:4.50%	0.368
	COIMBRA	6.80%	0.40%:13%	**0.021**
	Condeixa-a-Nova	5.70%	2.10%:9.20%	**0.002**
	Figueira da Foz	1.50%	-1.50%:4.60%	0.158
	Mealhada	-0.83%	-4.40%:3.10%	0.345
	Mira	8.70%	4.00%:13.00%	**0.001**
	Montemor-o-Velho	2.30%	-1.10%:5.80%	0.091
	Mortágua	5.70%	2.40%:9.10%	**0.002**
	Penacova	10.00%	6.70%:14.00%	**0.001**
	Soure	8.90%	4.30%:13.00%	**0.001**
BV	Águeda	1.90%	-0.81%:4.50%	0.091
	Albergaria-a-Velha	-0.86%	-4.00%:2.20%	0.286
	Anadia	12.00%	9.40%:16.00%	**0.001**
	Aveiro	-1.70%	-4.50%:1.10%	0.120
	Estarreja	1.20%	-2.80%:4.80%	0.286
	Ílhavo	2.20%	-0.76%:5.10%	0.079
	Murtosa	1.30%	-6.30%:7.90%	0.351
	Oliveira do Bairro	-4.40%	-8.10%:-1.20%	**0.006**
	Ovar	2.20%	-1.10%:5.70%	0.107
	Sever do Vouga	2.20%	-1.30%:5.60%	0.085
	Vagos	-2.60%	-5.50%:0.28%	**0.049***
CB	Belmonte	-0.73%	-7.40%:5.70%	0.419
	Covilhã	7.30%	3.50%:11.00%	**0.002**
	Fundão	4.90%	0.49%:9.00%	**0.017**
DL	Aguiar da Beira	7.30%	3.30%:11.00%	**0.002**
	Carregal do Sal	8.60%	3.40%:13.00%	**0.001**
	Castro Daire	0.74%	-3.60%:4.90%	0.357
	Mangualde	0.20%	-320%:3.60%	0.467
	Nelas	-10.00%	-15.00%:-5.50%	**0.001**
	Oliveira de Frades	8.60%	2.60%:14.00%	**0.006**
	Penalva do Castelo	11.00%	5.90%:16.00%	**0.002**
	Santa Comba Dão	1.10%	-2.70%:5.00%	0.291
	São Pedro do Sul	6.60%	2.80%:10.00%	**0.001**
	Sátão	-2.40%	-7.30%:1.90%	0.123
	Tondela	0.64%	-2.70%:3.90%	0.351
	Vila Nova de Paiva	12.00%	6.40%:17.00%	**0.002**
	Viseu	-0.49%	-3.90%:2.70%	0.376
	Vouzela	0.22%	-5.80%:5.90%	0.475
PIN	Alvaiázere	-5.20%	-9.80%:-0.70%	**0.013**
	Ansião	6.20%	1.60%:10.00%	**0.006**
	Arganil	17.00%	13.00%:21.00%	**0.001**
	Castanheira de Pera	-4.10%	-12.00%:3.90%	0.161
	Figueiró dos Vinhos	3.80%	-1.30%:8.90%	0.076

Sub-regions	Municipality	Relative Effect	95% CI	p-value
	Góis	15.00%	9.90%:19.00%	**0.004**
	Lousã	2.00%	-1.10%:5.10%	0.111
	Miranda do Corvo	5.70%	1.80%:9.30%	**0.002**
	Oliveira do Hospital	5.30%	1.50%:9.20%	**0.006**
	Pampilhosa da Serra	0.07%	-3.90%:4.00%	0.497
	Pedrógão Grande	2.60%	-2.70%:8.10%	0.165
	Penela	5.10%	-0.03%:11.00%	**0.030***
	Tábua	3.40%	-0.91%:7.60%	0.070
	Vila Nova de Poiares	6.50%	2.40%:11.00%	**0.005**
PL	Batalha	4.40%	0.84%:7.70%	**0.012**
	Leiria	-2.00%	-4.90%:1.10%	0.102
	Marinha Grande	-1.10%	-4.30%:1.90%	0.233
	Pombal	1.10%	-1.40%:3.80%	0.181
	Porto de Mós	-7.00%	-10.00%:-3.90%	**0.001**
PIS	Castelo Branco	-2.80%	-6.30%:0.46%	0.055
	Idanha-a-Nova	14.00%	8.00%:20.00%	**0.002**
	Penamacor	-4.00%	-9.80%:1.90%	0.096
	Vila Velha Ródão	-4.00%	-9.30%:1.70%	0.085
	Oleiros	0.79%	-7.50%:8.20%	0.423
	Proença a Nova	7.90%	2.30%:13.00%	**0.002**
	Sertã	-0.42%	-4.70%:3.40%	0.407
	Vila de Rei	7.10%	0.53%:14.00%	**0.019**
G	Almeida	37.00%	32.00%:42.00%	**0.002**
	Celorico Beira	20.00%	14.00%:25.00%	**0.002**
	Fornos Algodres	1.80%	-3.50%:7.10%	0.253
	Figueira Castelo Rodrigo	11.00%	6.10%:17.00%	**0.001**
	Gouveia	3.90%	-0.79%:8.30%	0.059
	Guarda	4.30%	0.38%:8.20%	**0.019**
	Manteigas	19.00%	14.00%:25.00%	**0.001**
	Meda	-11.00%	-17.00%:-4.20%	**0.003**
	Pinhel	14.00%	9.60%:19.00%	**0.001**
	Sabugal	7.30%	1.10%:13.00%	**0.012**
	Seia	3.50%	-0.40%:7.70%	**0.037***
	Trancoso	11.00%	4.50%:17.00%	**0.002**
	Vila Nova de Foz Côa	11.00%	1.40%:20.00%	**0.012**

95%CI: 95% credible interval; BM: Baixo Mondego; BV: Baixo Vouga; CB: Cova da Beira; DL: Dão Lafões; PIN: Pinhal Interior Norte; PL: Pinhal Litoral; BIS: Beira Interior Sul; PIS: Pinhal Interior Sul; G: Guarda.

*Note: In some municipalities, the relative effect 95%CI includes the zero, but the p-value (representing the posterior probability that the intervention had any effect) is significant. This is because the posterior probability is expressed in terms of a one-sided p-value, while the posterior interval for the effect corresponds to a two-sided hypothesis test. This occurs in municipalities where the relative effect was close to null.
